# Potential use of high-throughput sequencing of soil microbial communities for estimating the adverse effects of continuous cropping on ramie (*Boehmeria nivea* L. Gaud)

**DOI:** 10.1371/journal.pone.0197095

**Published:** 2018-05-11

**Authors:** Siyuan Zhu, Yanzhou Wang, Xiaomin Xu, Touming Liu, Duanqing Wu, Xia Zheng, Shouwei Tang, Qiuzhong Dai

**Affiliations:** Institute of Bast Fiber Crops, Chinese Academy of Agricultural Sciences, Changsha, Hunan, P.R. China; Institute for Sustainable Plant Protection, C.N.R., ITALY

## Abstract

Ramie (*Boehmeria nivea* L. Gaud) fiber, one of the most important natural fibers, is extracted from stem bark. Continuous cropping is the main obstacle to ramie stem growth and a major cause of reduced yields. Root-associated microbes play crucial roles in plant growth and health. In this study, we investigated differences between microbial communities in the soil of healthy and continuously cropped ramie plants, and sought to identify potential mechanisms whereby these communities could counteract the problems posed by continuous cropping. Paired-end Illumina MiSeq analysis of 16S rRNA and ITS gene amplicons was employed to study bacterial and fungal communities. Long-term monoculture of ramie significantly decreased fiber yields and altered soil microbial communities. Our findings revealed how microbial communities and functional diversity varied according to the planting year and plant health status. Soil bacterial diversity increased with the period of ramie monoculture, whereas no significant differences were observed for fungi. Sequence analyses revealed that *Firmicutes*, *Proteobacteria*, and *Acidobacteria* were the most abundant bacterial phyla. *Firmicutes* abundance decreased with the period of ramie monoculture and correlated positively with the stem length, stem diameter, and fiber yield. The *Actinobacteria*, *Chloroflexi*, and *Zygomycota* phyla exhibited a significant (P < 0.05) negative correlation with yields during continuous cultivation. Some *Actinobacteria* members showed reduced microbial diversity, which prevented continuous ramie cropping. *Ascomycota*, *Zygomycota*, and *Basidiomycota* were the main fungal phyla. The relatively high abundance of *Bacillus* observed in healthy ramie may contribute to disease suppression, thereby promoting ramie growth. In summary, soil weakness and increased disease in ramie plants after long-term continuous cropping can be attributed to changes in soil microbes, a reduction in beneficial microbes, and an accumulation of harmful microbes.

## Introduction

Ramie (*Boehmeria nivea* L. Gaud), also known as “China grass,” is a perennial, diploid (2n = 28), herbaceous plant that belongs to the *Urticaceae* family. Ramie fiber is naturally produced, can be extracted from stem bark, and possesses several excellent characteristics, such as long strands, smooth texture, and high tensile strength. In addition, ramie contains a high level of crude protein in its leaves and young stems, and is used for livestock forage [1]. Ramie must have a high vegetable yield to be useful for fiber and feed [2]. Ramie is one of the oldest fiber and feed crops in China, and is an important natural fiber crop in India and other Southeast Asian and Pacific Rim countries [3]. However, owing to problems arising from continuous cropping, ramie-cultivation areas have shrunk substantially in China, impairing the development of a viable ramie industry. Problems related to continuous cropping are widespread in agriculture, both in annual crops (such as cotton, cucumbers, and potatoes) and in perennial plants (such as apples, peaches, ramie, and sugarcane) [4–7].

Continuous cropping can cause soil-quality degradation, crop-yield reduction, and aggravated crop pest-induced plant diseases [8, 9]. Three factors determine problems associated with continuous cropping: an imbalance in soil nutrients, autotoxicity of root exudates, and shifts in the microbial community composition [10]. Crop rotation, fallow, and manure application have been commonly used in agriculture to overcome these issues [11–13] as they can improve the soil microstructure, even if only temporarily [14–15]. Soil microorganisms are extremely important for maintaining soil health, which is fundamental for plant production in agricultural systems [16–18]. Data from previous studies revealed that soil microbial communities were affected by various factors, including the plant species, soil type, and agricultural management [19]. Several recent studies have shown that continuous cropping results in disruption of the soil microbial community composition and structure [20–22]. An increase in fungal pathogens and simplification of the beneficial fungal community was reported to cause decreased peanut growth and yields over many years of continuous cropping [23]. Soil weakness and vanilla stem wilt disease can be attributed to alterations in the soil fungal community, reduced levels of beneficial microbes, and an accumulation of soil-borne *Fusarium* pathogen spp. after long-term continuous vanilla cropping [24]. Comparatively less bacterial diversity was observed in apple rhizosphere soil (RS) when planted in a replant site than in a new site [25]. Analyses of 16S ribosomal RNA (rRNA) genes in tea orchard soils revealed that soil bacterial community numbers and structures were significantly affected by the duration of continuous cropping [26]. In summary, a healthy and stable soil microstructure is essential for maintaining long-term continuous cropping and stable high crop yields. However, the detailed effects of long-term continuous cropping on soil microbial communities and their links to soil sickness remain unclear [27].

Previous studies of soil microbial communities under continuous cropping relied mostly on the construction of 16S rRNA and 18S rRNA gene libraries and performing denaturing gradient gel electrophoresis; however, only certain dominant microbial groups can be detected using these methods [27, 23]. Recent next-generation sequencing (NGS) methods, such as 454 pyrosequencing and Illumina-based techniques, may provide a more direct way of detecting microbial taxa, especially those with low-abundance species changes [28, 29]. In addition, Illumina sequencing is cost-effective and can yield 10 times or more sequences per sample than 454 pyrosequencing, thereby enabling the analysis of a high number of detailed taxonomic profiles [30]. To ascertain the relationship between rhizosphere microorganisms and impaired growth arising from continuous ramie cropping, and to promote the sustainable development of the ramie industry, we employed for the first time an Illumina-based NGS approach to profile the main microbial rhizosphere taxa and functional diversity associated with ramie plants grown in a field subjected to continuous ramie cropping.

## Materials and methods

### Ethics statement

This work was conducted in our scientific research field for ramie cultivation studies, which is owned by our institution. Therefore, no specific permissions were required for these using these locations or performing the study. The field studies did not involve endangered or protected species.

### Site description and sample collection

The experimental site was located in Yuanjiang, Hunan Province, China (112° 33' E, 28° 16' E), which is a main cultivation region for ramie. The cultivar of the planted ramie (Zhongzhu No. 1), agronomic management, and fertilization regime were similar between the 1-year and 10-year continuously cropped ramie cultivations. In May 2016, soil samples were collected from both time-series ramie fields. Soil that adhered tightly to the root system was collected as RS. Soil that was not attached tightly to the root system was defined as non-rhizosphere soil (NRS). RS and NRS samples were obtained from 1-year healthy ramie fields (1YR and 1YN, respectively) and 10-year continuous-cropping ramie fields (10YR and 10YN, respectively). For the 1-year and 10-year planting fields, we randomly collected RS after removing the ramie plants, and the surface coverings from 15 plants were mixed to comprise 1 sample. NRS was also collected from 15 plants to comprise 1 sample. Each field was sampled in triplicate. Each sample was sifted through a 2-mm sieve and was thoroughly homogenized. Two copies of each sample were analyzed, and 1 sample was kept in case additional testing was needed. In total, 12 soil samples were placed into separate sterile plastic bags and stored at –70°C for subsequent DNA extraction in our laboratory.

### Soil DNA extraction

For each sample, total genomic DNA was extracted using the E.N.Z.A. Soil DNA Kit (Omega Bio-tek), following the manufacturer’s instructions. The quality and quantity of DNA was verified using a NanoDrop spectrophotometer and by agarose gel electrophoresis. Extracted DNA was diluted to 1 ng/μl and stored at –20°C until further processing.

### PCR amplification and Illumina high-throughput sequencing analysis

Diluted DNA from each sample was used as a template for PCR amplification of bacterial 16S and fungal ITS rRNA gene sequences with barcoded primers and HiFi Hot Start Ready Mix (KAPA). For bacterial-diversity analysis, the variable V3–V4 regions of the 16S rRNA genes were amplified using the universal primers 338F (5'-ACTCCTACGGGAGGCAGCAG-3') and 806R (5'- GGACTACHVGGGTWTCTAAT-3') [31]. For fungal-diversity analysis, the ITS2 variable regions were amplified using the universal primers fITS7 (5'- GTGARTCATCGAATCTTTG-3') [32] and ITS4 (5'-TCCTCCGCTTATTGATATGC-3') [33]. The amplicon quality was assessed by visualization after gel electrophoresis, followed by purification with AMPure XP beads (Agencourt), and amplification in another round of PCR. After a second round of purification with the AMPure XP beads, the final amplicon was quantified using the Qubit dsDNA Assay Kit. Equal amounts of purified amplicon were pooled for subsequent sequencing.

High-throughput sequencing of amplicons was performed using the Illumina Mi-Seq platform (Illumina, San Diego, CA, USA) at Oebiotech Company (Shanghai, China). Complete data sets generated in this study were deposited in the NCBI Sequence Read Archive database under accession numbers SRP110777 and SRP110558. Raw paired-end reads were subjected to quality filtering using Trimmomatic software before paired-end read assembly was performed with FLASH software [34]. Sequences were analyzed with QIIME software (version 1.8.0) [35] and the UPARSE pipeline [36]. The UPARSE pipeline was then used to detect operational taxonomic units (OTUs) at 97% similarity. A representative sequence was selected for each OTU and used to assign the taxonomic composition with the RDP classifier [37].

### Statistical analysis

For all parameters, multiple comparisons were performed using one-way analysis of variance with Turkey's honest significant difference multiple-range test. For α-diversity, all analyses were based on OTU clusters, with a cutoff of 3% dissimilarity. The Chao1 index was calculated to estimate the richness of each sample. Diversity within each sample was estimated using the nonparametric Shannon diversity index. Rarefaction curves based on the average number of observed OTUs were generated using the Mothur software package to compare the relative levels of bacterial and fungal OTU diversity across continuous-cropping ramie field soil samples. For β-diversity, hierarchical cluster dendrograms (with Bray–Curtis distance dissimilarities) were performed using Mothur, based on the OTU composition. The dendrograms were used to compare bacterial and fungal community structures across all soil samples. Heatmaps and Venn diagrams were generated using custom R scripts. Weighted and unweighted UniFrac distance metrics (based on the phylogenetic structure) [38] were used to generate principle coordinate-analysis plots to further assess the similarities between the community compositions of different samples. Pearson correlation coefficients were calculated using SPSS 22.0 statistical software (Chicago, IL, USA). Histograms were created in Microsoft Excel 2010.

## Results

### Ramie stem traits and fiber yields in response to continuous cropping

**Fig 1:**
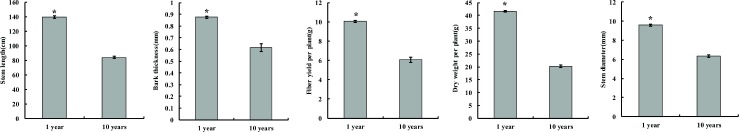
Changes in ramie fiber yield and stem traits in response to continuous cropping. Note: The error bars represent the standard error, and * represent significance at *P* < 0.05.

In 1-year cultivated ramie, the stem length, diameter, and bark thickness were 139.3 cm, 9.59 mm, and 0.875 mm, respectively (Fig 1). A significant decrease in the stem length, diameter, and bark thickness (83.9 cm, 6.35 mm, and 0.615 mm, respectively) was observed after 10 years (Fig 1). In addition, the fiber yield per plant was higher in 1-year ramie (10.05 g) than in 10-year ramie (6.07 g), as was the dry weight per plant (41.62 g and 20.17 g, respectively) (Fig 1).

### Overall diversity of microbial communities in 2 time-series ramie fields

Differences in rarefaction curves were observed between microbial communities derived from 1-year healthy and 10-year continuous-cropping ramie RS and NRS. A total of 670,111 high-quality sequences (303,739 for bacteria and 366,372 for fungi) were obtained after filtering low-quality reads, chimeras, and attachment sequences. Clean sequence reads included 85,918 for 1YR bacteria, 43,095 for 10YR bacteria, 67,839 for 1YR fungi, and 80,267 for 10YR fungi (Table 1).

**Table 1:** Illumina Miseq reads, number of operational taxonomic units (OUTs), and alpha diversity in healthy and continuous cropping ramie.

Microbial community	Sample	Reads		No. of OTUs	Observed species	Alpha diversity	
		Raw	Clean			Shannon	Chao1
Bacteria	1YR	99543	91653	15771	2086	8.47	3085
	1YN	129645	119129	14842	1740	6.73	2707
	10YR	48183	43095	10168	1982	8.24	2913
	10YN	58210	55597	7333b	1334	7.22	2050
Fungi	1YR	71555	67839	1141	371	5.89	430
	1YN	123306	122689	860	256	4.83	286
	10YR	84648	80267	1112	458	6.38	518
	10YN	119814	95577	969	287	4.27	360

**Fig 2:**
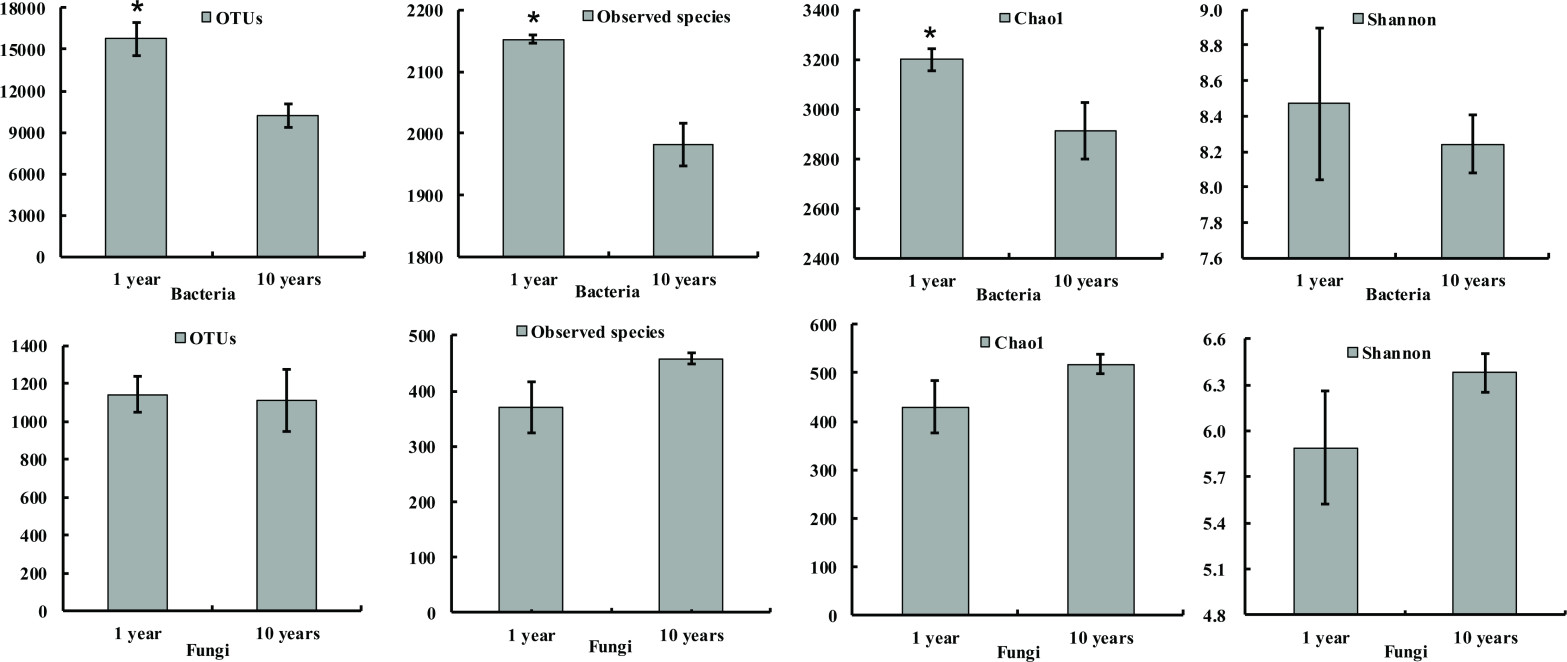
Comparison of OTUs and microbial (bacterial and fungal) diversity between the 1-year ramie soil and 10-year continuous-cropping ramie soil. Note: * represents significance at *P* < 0.05.

The total number of OTUs detected at 97% shared sequence similarity was very high in ramie RS, both in terms of bacteria and fungi, and the estimated α-diversities indicated abundant microbial diversity was present in all samples (Table 1). For bacteria, the number of different phylogenetic OTUs ranged from 7,333 to 15,771, with 1-year healthy ramie (1YR, 1YN) showing higher 16S rRNA gene diversity than 10-year continuously cropped ramie (10YR, 10YN). 1YR samples presented the highest number of OTUs and bacterial diversity, whereas 10YN samples had the lowest. For fungi, the number of different phylogenetic OTUs in all samples ranged from 969 to 1,141, with 1-year healthy ramie exhibiting higher diversity than 10-year continuously cropped ramie. The 10YR samples displayed the highest Shannon index and number of OTUs, whereas the 1YN samples had the lowest OTU number (Table 1). Statistical analysis of OTUs and microbial (bacterial and fungal) diversity are shown in Fig 2. In bacteria, the results showed that the OTUs, number of observed species, and Chao1 index of 1-year-old ramie soil were obviously higher than that in 10-year-old ramie soil, whereas no obvious differences in those parameters were noticed in fungi (Fig 2).

### Microbial community composition in soil with continuous ramie cultivation

**Fig 3:**
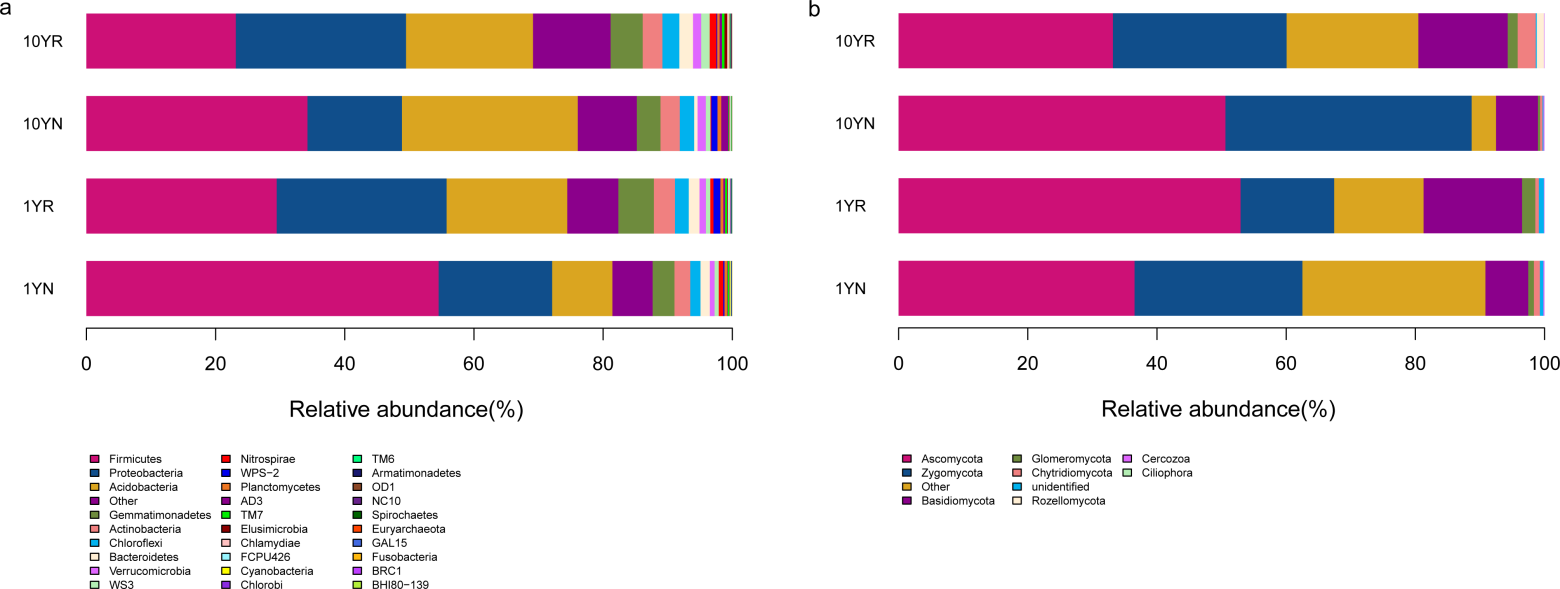
**Relative abundances of the dominant bacterial (a) and fungal (b) taxa in 1-year and 10-year continuous-cropping ramie soil samples at the phylum level.** which were identified using the RDP classifier. Sequences not classified into any known group were designated as “Other”.

All valid reads were classified from the phylum to the genus level using the default settings in QIIME. The overall microbial compositions of the samples were similar; however, different proportions were observed for some samples at the phylum level (Fig 3). Based on bacterial phylum-assignment results (Fig 3A), high-throughput sequencing produced few unclassified reads and, on average, 93.70% of bacterial 16S rRNA sequence reads were classified as members of 29 different phyla. *Firmicutes* was the most abundant phylum in all samples, accounting for 23.13–54.53% of all valid reads. Significantly more *Firmicutes* taxa were detected in 1-year (1YR, 1YN) than in 10-year (10YR, 10YN) cropping soil samples. *Proteobacteria* was the second most abundant phylum, with an average relative abundance of 21.21%. Other dominant phyla included *Acidobacteria* (average of 18.72%), *Gemmatimonadetes* (4.38%), *Actinobacteria* (2.94%), *Chloroflexi* (2.14%), and *Bacteroidetes* (1.4%). Fungal-classification results showed that the dominant phylum was *Ascomycota*, accounting for 33.19–52.96% of all valid reads, with an average relative abundance of 43.32%. The next most dominant phyla were *Zygomycota* (26.37%), *Basidiomycota* (10.54%), *Glomeromycota* (1.21%), and *Chytridiomycota* (1.16%). Compared with 10-year continuous-cropping ramie soil (10YR, 10YN), the 1-year samples (1YN, 1YR) contained higher percentages of *Zygomycota* (32.05 vs. 20.69%, respectively), but relatively lower percentages of *Basidiomycota* (6.55 vs. 14.54%) (Fig 3B).

### Differences in microbial communities between healthy and continuously cropped ramie samples

**Fig 4:**
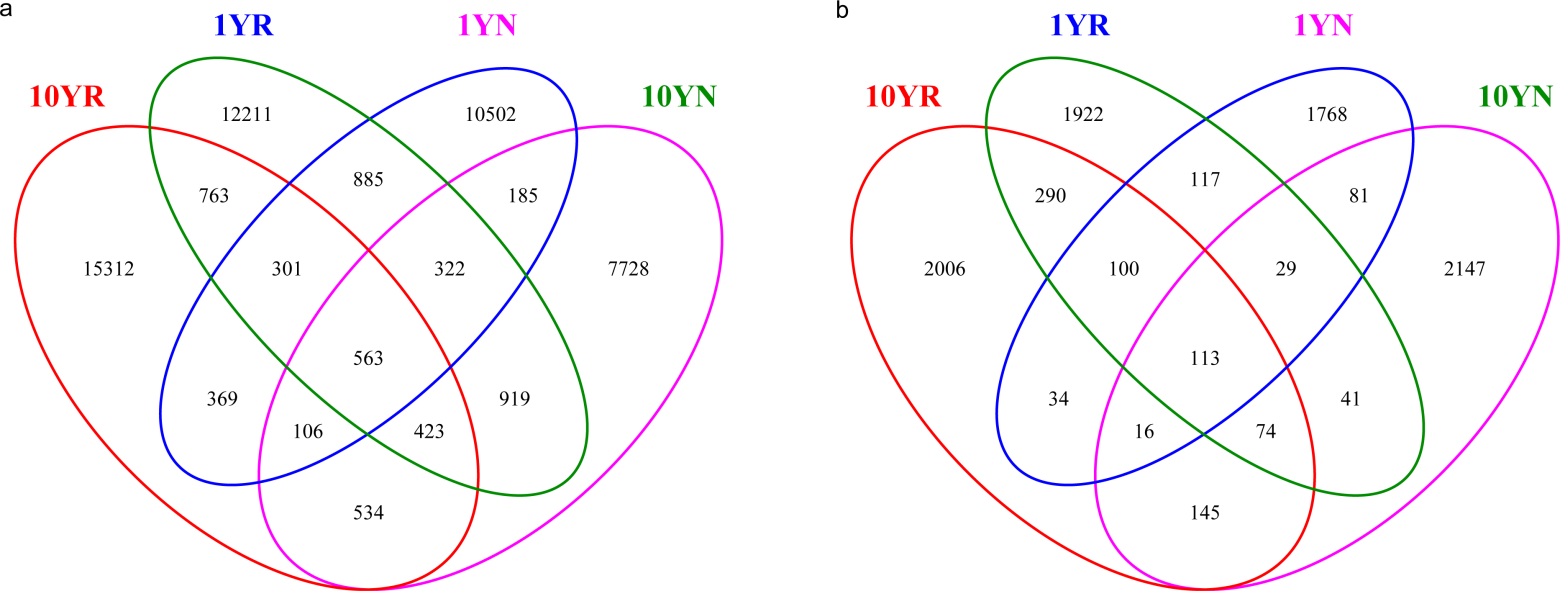
Venn diagram showing the number of unique bacterial (a) and fungal OTUs (b) detected in ramie soil samples.

Venn diagrams were generated using the Mothur program, based on the shared OTU tables from 4 different soil samples (Fig 4). The total number of unique bacterial OTUs was 51,123, of which 885 were associated only with 1-year healthy cropping ramie (1YN, 1YR), 534 were associated only with 10-year continuously cropped ramie (10YN, 10YR), and 563 were shared by all samples (Fig 4A). At the genus level, the OTUs of 1-year healthy cropping ramie (1YN, 1YR) included *Bacillus*, *Bdellovibrio*, *Aquicella*, *Nitrospira*, *Lactococcus*, and *Paracoccus*, whereas *Sphingomonas*, *Arthrobacter*, *Cytophaga*, *Burkholderia*, *Flavobacterium*, *TM7-1*, and *Phenylobacterium* were detected in 10-year continuous-cropping ramie samples (10YN, 10YR). In terms of fungi, 8,883 different OTUs were identified, of which 117 were associated only with 1-year healthy-cropping ramie soil, 145 were associated only with 10-year continuous-cropping ramie soil, and 113 were shared by all samples (Fig 4B). Among the fungal genera, *Cortinarius*, *Malassezia*, *Mortierella*, *Ceratobasidium*, *Guehomyces*, *Rhizophydium*, *Fusarium*, and *Trichoderma* were detected only in the 1-year healthy-cropping ramie soil, whereas *Leucosporidium*, *Sporobolomyces*, *Cryptococcus*, *Humicola*, and *Trichosporon* were detected only in 10-year continuous-cropping ramie soil.

**Fig 5:**
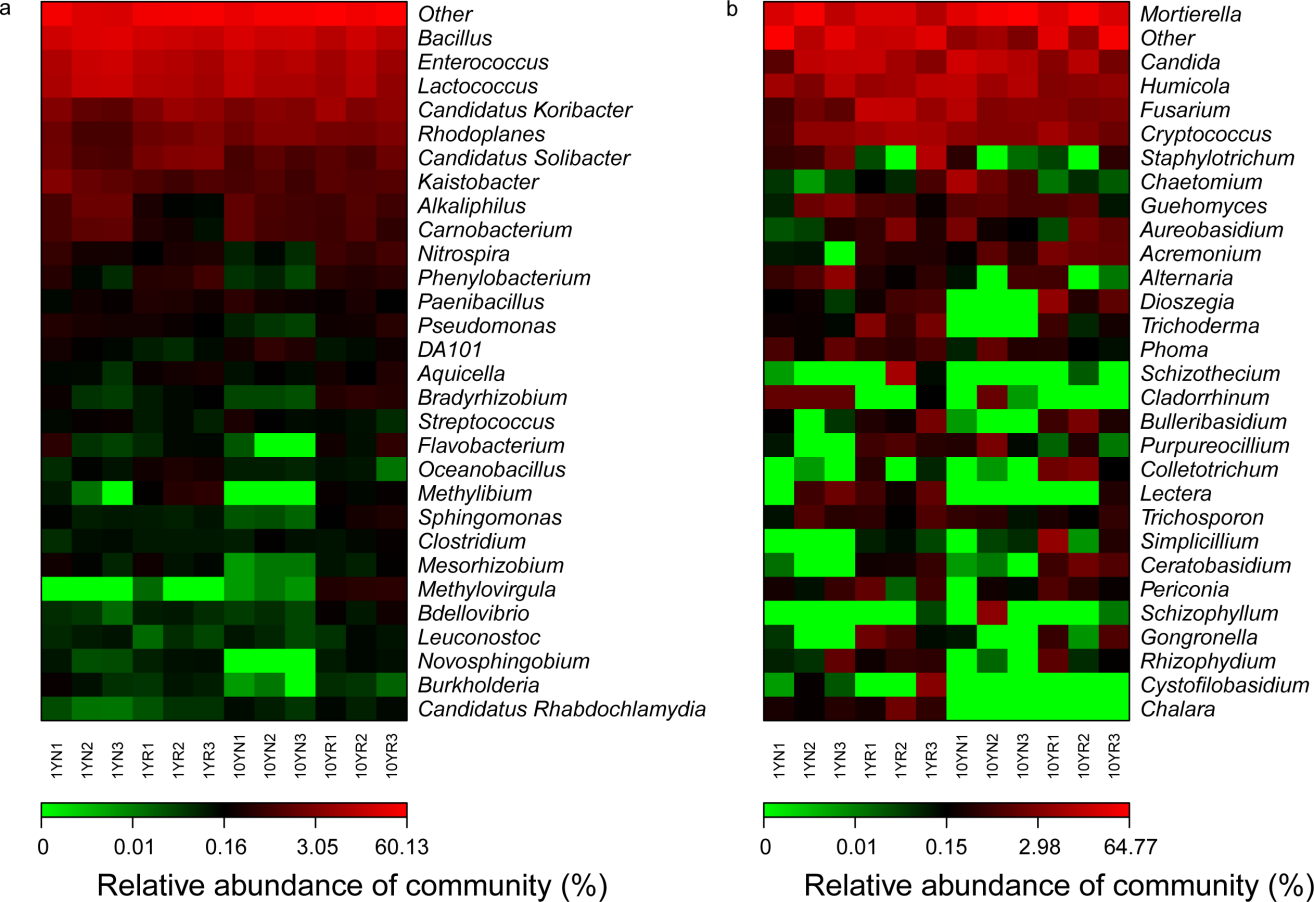
Heatmap analysis of bacteria (a) and fungi (b) based on the relative abundances of dominant genera from different Ramie soil samples.

Heatmap clustering analysis results for the 30 dominant genera from the different samples are shown in Fig 5. Among bacteria, the differences between 1-year healthy-cropping and 10-year continuous-cropping ramie samples were attributable mostly to *Bacillus*, *Enterococcus*, *Lactococcus*, *Candidatus Koribacter*, *Rhodoplanes*, “Candidatus” *Solibacter*, *Kaistobacter*, *Alkaliphilus*, and *Carnobacterium*. The *Methylovirgula* genera had high abundances in the RS of 1-year and 10-year ramie, *Alkaliphilus and Carnobacterium* genera had relatively high abundances in the RS of 10-year continuous-cropping ramie, but the *Methylibium* genus had a relatively high abundance in the RS of all 1-year and 10-year ramie. For fungi, heatmap clustering analysis showed that the *Mortierella*, *Candida*, *Humicola*, *Fusarium*, and *Cryptococcus* genera had relatively high abundances, and the *Bulleribasidium*, *Rhizophydium*, and *Gongronella* genera differed between 1-year healthy-cropping and 10-year continuous-cropping ramie samples. The *Ceratobasidium* and *Colletotrichum* genera had high abundances in the RS of 1-year and 10-year ramie, whereas the *Trichoderma*, *Lectera* and *Chalara* genera had low abundances in 10-year ramie.

**Fig 6:**
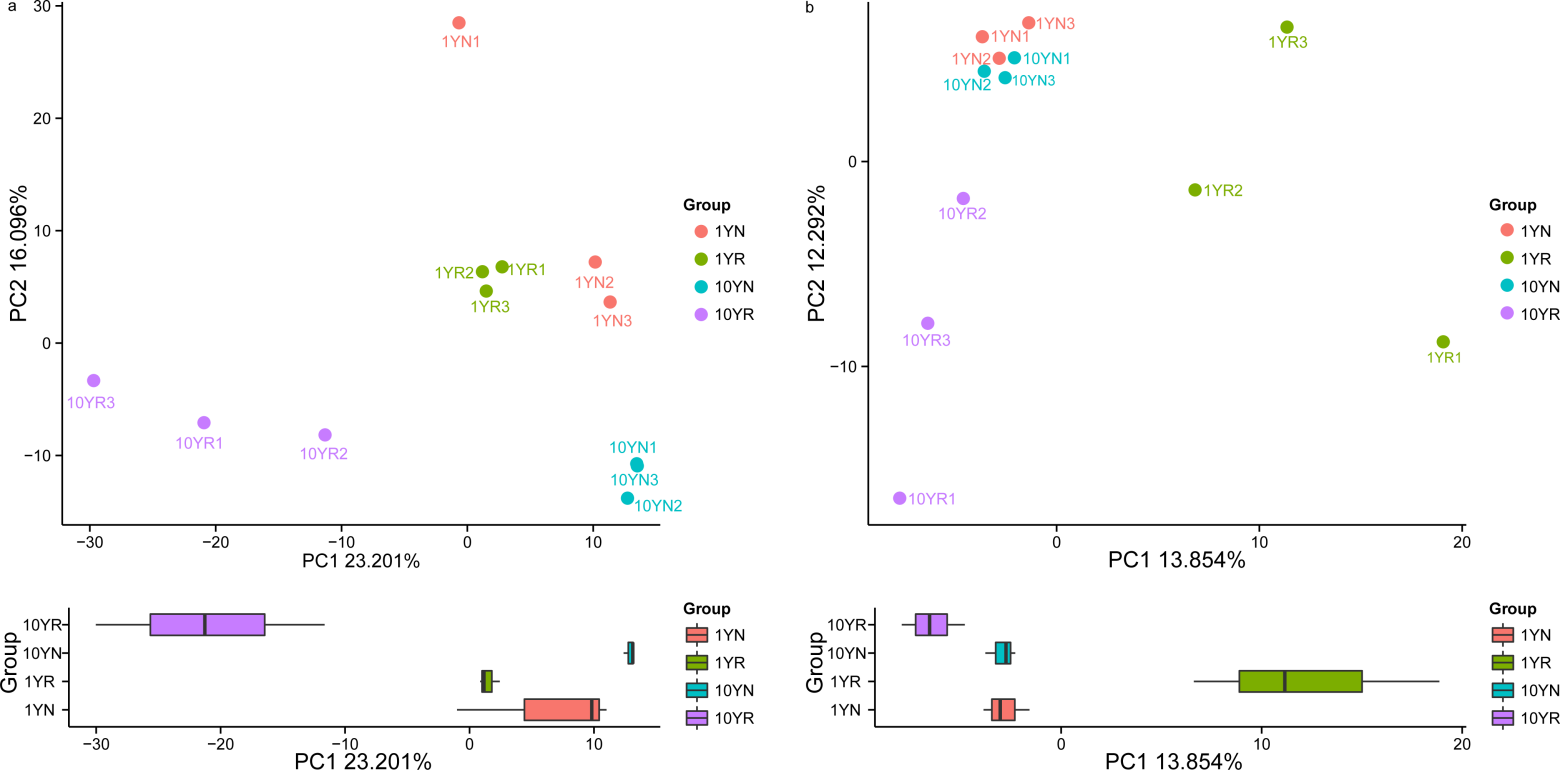
PCA of the OTUs detected major variations in the bacterial (a) and fungal (b) communities in healthy and continuous Ramie soil samples.

To further compare the microbiota among different samples, we performed principal component analysis (PCA) to study the relative abundances of bacterial and fungal genera, using Canoco software, version 4.5 (Fig 6). The data are presented as a 2-dimensional plot to better illustrate the relationship among these soil samples. In bacteria (except for 1YN.1), the 1YN and 1YR groups were relatively similar, indicating that communities in most samples shared similar diversity. The 10YR samples had a relatively higher PC1 value, whereas the 1YN samples had a higher PC2 value. In fungi, the 1YN and 10YN groups were similar, but the 1YR group had a relatively higher PC1 value. The PCA results agreed with those from the heatmap analysis, indicating that all samples exhibited different characteristic bacterial and fungal communities.

### Pearson correlation coefficients between yield and bacterial or fungal phyla

We used Pearson correlation coefficients to evaluate the relationships between abundant phyla (bacterial and fungal) and the yield properties of continuous-cropping ramie samples (Table 2). **Pearson’s correlation** **relationships between microbial (bacterial and fungal) phyla (RA >1%) and yield properties of continuous ramie.**The relative abundances of the *Firmicutes*, *Bacteroidetes*, and *Glomeromycota* phyla correlated positively with the yield properties of continuous ramie. In contrast, *Actinobacteria*, *Chloroflexi*, and *Zygomycota* exhibited a significantly (P < 0.05) negative correlation with the yield properties of continuous ramie. Other genera did not display any significant correlation (Table 2). **Pearson’s correlation** **relationships between microbial (bacterial and fungal) phyla (RA >1%) and yield properties of continuous ramie.** Thus, *Actinobacteria*, *Chloroflexi*, and *Zygomycota* appear to have exerted the largest impact on yields of continuous-cropping ramie.

**Table 2:** Pearson’s correlation relationships between microbial (bacterial and fungal) phyla (RA > 1%) and yield properties of continuous ramie.

Phyla	Stem Length	Stem Diameter	Bark thickness	Fiber Yield	Dry weight
*Firmicutes*	0.479	0.470	0.387	0.556	0.519
*Proteobacteria*	0.240	0.154	0.407	0.223	0.208
*Actinobacteria*	-0.723**	-0.605	-0.789**	-0.831**	-0.747**
*Gemmatimonadets*	0.169	0.148	0.278	0.090	0.126
*Actinobacteria*	-0.174	-0.113	-0.148	-0.306	-0.221
*Chloroflexi*	-0.697*	-0.708*	-0.558	-0.735*	-0.726*
*Bacteroidetes*	0.277	0.126	0.482	0.342	0.265
*Ascomycota*	0.174	0.341	0.006	0.009	0.147
*Zygomycota*	-0.800**	-0.751*	-0.874**	-0.765*	-0.777*
*Basidiomycota*	0.199	0.150	0.335	0.145	0.160
*Glomeromycota*	0.496	0.434	0.621	0.456	0.463
*Chytridiomycota*	-0.388	-0.539	-0.174	-0.284	-0.388

RA is relative abundance.

* and ** represent significance at *P* < 0.05 and 0.01, respectively.

## Discussion

Ramie is a perennial, herbaceous plant. Long-term continuous cropping of ramie results in reduced yield and even death. This phenomenon has been observed also for many other plants, including cotton [39], apple [25], and sugarcane [7], whose yields are significantly lower after long-term continuous cropping. Ramie fiber extracted from stem bark is a vegetative tissue whose yield is determined by stem growth. In this study, continuous cropping for 10 years severely inhibited stem growth and led to a decrease in fiber yield (Fig 1). Hence, a stable and healthy long-term growth environment is essential for maintaining high ramie production. Recently, as the planting area of ramie has decreased greatly, the economic benefit has become reduced. Problems linked to continuous cropping represent some of the main hindrances to the development of the ramie industry. Therefore, elucidating potential microbial community-structure mechanisms that could improve continuous cropping tolerance are highly important for ramie production in China. Illumina NGS technology is a powerful tool in many research areas, including re-sequencing, micro-RNA expression profiling, DNA methylation, *de novo* transcriptome sequencing, and whole-genome sequencing [40–42]. In this study, NGS was used to identify the microbial community associated with ramie plants grown under continuous field conditions and to elucidate differences in community structure based on the health status of plants. Limited studies on the interactions between continuous cropping ramie and the rhizospheric microbial community have been conducted in natural ecosystems. To the best of our knowledge, this study represents the first implementation of Illumina sequencing technology to investigate the microbial diversity associated with healthy and continuous RS samples from ramie plants grown under field conditions.

Interestingly, we analyzed the diversity of ramie rhizospheric soil according to richness (Chao 1) and diversity (Shannon) indices, which showed marked changes between 1-year healthy cropping ramie and 10-year continuous-cropping ramie, whereas no obvious change in fungi was observed (Fig 2 and Table 1). The fiber yield of 10-year continuous ramie decreased significantly compared to that of 1-year ramie. Thus, we speculated that changes in the bacterial composition and diversity were more related to the continuous cropping of ramie. Moreover, these properties varied according to the planting year and health status of the ramie plants. The community structure of soil microorganisms underwent changes during different years of ramie cultivation, resulting mainly in reduced microbial diversity. This trend was analogous to that reported for *Picea mariana* based on Sanger sequencing, which showed that healthy seedlings grown in a nursery had greater bacterial diversity than that of diseased seedlings [43]. Healthy greenhouse tomato RS samples have been reported to harbor higher bacterial diversities than RS samples from diseased tomatoes, according to 454 pyrosequencing results [44]. However, in studies based on Illumina sequencing, healthy Lanzhou lily (*Lilium davidii*) plants exhibited lower bacterial diversity than diseased plants [45]. Differences in plant-growth conditions, plant disease-causing agents, microbe-identification methods, and plant genotypes may explain these contrasting results.

In this study, sequence analyses revealed that the relative abundances of bacterial and fungal phyla (Fig 3). For bacteria, we observed that *Firmicutes*, *Proteobacteria*, and *Acidobacteria* were the most abundant bacterial phyla in 1-year healthy and 10-year continuous ramie (Fig 3A). The most dominant phylum was *Firmicutes*. Data from numerous studies have shown that most rhizosphere species belong to the *Firmicutes* phylum [46–48] Moreover, *Firmicutes* correlated positively with the stem length, stem diameter, and fiber yield of continuous ramie (Table 2), and obvious differences were observed in the *Firmicutes* phylum in the RS and NRS between 1-year healthy and 10-year continuous ramie. This change may represent the main factor causing soil weakness after continuous ramie cropping, as reported previously [45, 49]. *Firmicutes* was implicated as serving a very important role in the growth and yield of ramie, but the underlying mechanism needs to be determined in a future study.

The second-most dominant phylum was *Proteobacteria*. Although no obvious differences were observed in RSs between 1-year healthy and 10-year continuous ramie (Fig 3A), an obvious difference was found between RS and NRS from ramie. *Proteobacteria* have been previously shown to be enriched in cucumber rhizosphere [50, 6]. Recent studies of maize [51], soybeans [52], oak trees [53], and poplar trees [54] have also revealed an enrichment of *Proteobacteria* in the rhizosphere. The other independent studies have shown that *Proteobacteria* are dominant members of the rhizosphere microbiota [55–56]. These findings are in line with *Proteobacteria* having a fast-growth phenotype among rhizosphere bacteria and being capable of utilizing a broad range of root-derived carbon substrates [57].

We found that the relative abundance of *Proteobacteria* was elevated in ramie rhizosphere. Thus, we speculated that some species of *Proteobacteria* may serve as beneficial bacteria agents in the rhizosphere of ramie, some ones may beneficial for ramie growth. Our results showed that *Actinobacteria* were the most sensitive in the major distribution zone of ramie roots, and a significant negative correlation was found between *Actinobacteria* abundance and the stem length, stem diameter, and fiber yield. Some *Actinobacteria* showed reduced microbial diversity and, thus, may correlate with the adverse effects of continuous ramie cropping. This finding suggests that ramie cultivation strongly influences the community structure of soil *Actinomycetes*.

Previous findings have suggested that changes in the soil microflora may be responsible for impairing the growth of some continuously cropped plants [57]. Among microorganisms, some fungi have been found to play important roles in soil ecosystems and contribute to plant diseases [58]. Among fungi, *Ascomycota*, *Zygomycota*, and *Basidiomycota* were identified as the main phyla of ramie soil, which was consistent with previous continuous-cropping studies of peanut and soybean soils [22, 59]. Moreover, these phyla may be involved in the degradation of simple root exudates, as well as the more complex compounds present in sloughed root cells [60].

With an increase in the number of continuous planting years, reduced crop production and other barriers may appear, and a substantial body of research has shown that rhizosphere microbial diversity can be partially influenced by the cropping history [61]. In our study, the abundance of *Ascomycota* decreased in the RS of 10-year ramie, and the opposite results were found in NRS. *Ascomycota* also changed with increasing numbers of years of ramie cropping, and the microbial population densities in the rhizosphere were much higher than in the surrounding bulk soil. This phenomenon is known as the “rhizosphere effect” [62]. We speculate that it is likely that the rhizosphere effect led to a decrease of some beneficial *Ascomycota* genera or increased the abundance of some harmful genera in the 10-year RS of ramie. *Ascomycota* have function in the decay of organic substrates and act as mutualists [63]. Thus, we speculate that the *Ascomycota* genera may also function in the decay of organic substrates in the ramie rhizosphere.

In soil ecosystems, rhizosphere microorganisms interact with plants [64] and are escorted by a myriad of microorganisms living freely or in intimate association with their roots, which leads to root and stem development, growth stimulation, or crown rot diseases [65]. Some *Glomeromycota* members have been considered generally as obligate symbiotic fungi, and the *Ascomycota* and *Glomeromycota* phyla can respond rapidly to rhizodeposits [66, 67]. Previous data showed that members of the *Glomeromycota* phylum depend on carbon and energy derived from plant synthesis to survive, and shares a symbiotic relationship with the roots of plants [68, 69]. Therefore, we propose that the *Glomeromycota* phylum may also affect symbiosis and interactions between ramie roots and soil microbes, considering that it was highly enriched in the ramie rhizosphere. Soil fungi show high functional diversity. *Chytridiomycota* was abundant in the RS of 10-year ramie soil, which may be a result of continuous cropping, single nutrient supply, and conditions that augment the growth of harmful soil microbes [70].

At the genus level (Fig 5), among the bacteria identified in this study, *Bacillus*, *Aquicella*, *Lactococcus*, and *Paracoccus* were more abundant in 1-year healthy ramie, whereas *Cytophaga*, *Burkholderia*, *Flavobacterium*, *TM7-1*, and *Phenylobacterium* were more frequent in 10-year continuously cropped ramie. Among these genera, *Bacillus* is one of the most common plant growth-promoting rhizobacteria biocontrol agents [71]. Members of the *Flavobacterium* genus participate in mineralizing various types of organic matter. *Bacillus* and *Lactococcus* are members of the *Firmicutes* phylum, which was more abundant in healthy 1-year ramie soil. The abundance of the *Bacillus* genus decreased with the number of years of continuous cropping, and this genus was not observed after 10 years. Many previous findings have demonstrated that *Bacillus* plays important roles in the health of vanilla plants [72] and in the suppression of soil-borne diseases [73], indicating that the decrease in beneficial bacterial species may cause soil weakness after long-term ramie monoculture. Accordingly, we believe that the relatively high abundance of *Bacillus* among *Firmicutes* in healthy RS may contribute to disease suppression, thereby promoting ramie growth. Results from this study demonstrated that *Mortierella*, *Fusarium*, and *Phoma* were the major harmful genera in the 10-year continuously cropped ramie (Fig 5), further illustrating that the accumulation of harmful microorganisms could impair continuous ramie cropping, which is consistent with previous results [74]. For example, the main pathogens of wilt diseases in the Lanzhou lily field were members of the *Fusarium* genus [75]. The community structure of soil microorganisms is mainly determined by environmental factors such as soil properties [76], as well as the crop type and tillage-management measures [22, 23]. Analysis of ramie RS and NRS showed that the contents of *Alkaliphilus and Carnobacterium* genera were relatively high in 10-year ramie and that the *Methylovirgula* and *Methylibium* generas were more abundant in 1-year and 10-year ramie. Therefore, these genera may play relatively important roles for ramie roots.

Among the fungi identified in our samples, *Cortinarius*, *Fusarium*, and *Trichoderma* were relatively more abundant in healthy ramie, whereas *Cryptococcus* and *Trichosporon* were more abundant in continuously cropped ramie. The latter are major soil pathogens and may be one of the main causes of the adverse growth effects induced by continuous ramie cropping.

In conclusion, continuous ramie cropping led to a significant decline in stem length, stem diameter, and fiber yield. The soil microbial community composition was altered following long-term continuous cropping of ramie. Soil bacterial diversity decreased with the time span of ramie monoculture, whereas no significant differences were observed for fungi (Fig 2). We speculate that the relatively high abundance of *Bacillus* among *Firmicutes* in healthy RS may contribute to disease suppression, thereby promoting ramie growth. In general, soil weakness and ramie disease increased after long-term continuous cropping, and both conditions can be attributed to alterations in soil microbes, a reduction of beneficial microbes, and the accumulation of harmful microbes. The dominant bacterial and fungal phyla/genera are probably responsible for the growth inhibition resulting from continuous cropping of ramie. Furthermore, this is the first report to provide evidence of major differences between the microbial communities in the soil of healthy and continuously cropped ramie plants. Our results demonstrate that Illumina NGS technology was a useful and effective approach for characterizing the microbial communities of ramie RS. These differences in microbes provide important information regarding beneficial ramie rhizosphere microbial species, which can contribute to future improvements in the microbial community structure and decreasing pathogenic microbial diseases associated with continuously cropped ramie soil.
